# Supplementary material for: Enhancement of Rydberg-mediated single-photon nonlinearities by electrically tuned Förster resonances
Source: Nat Commun. 2016 Aug 12;7:12480. doi: 10.1038/ncomms12480 (PMC4990648; doi:10.1038/ncomms12480)
Supplement: Supplementary Information — Supplementary Note 1 and Supplementary References [file ncomms12480-s1.pdf]

Supplementary Information

H. Gorniaczyk,[1,*] C. Tresp,[1] P. Bienias,[2] A. Paris-Mandoki,[1] W. Li,[3]
I. Mirgorodskiy,[1] H. P. Büchler,[2] I. Lesanovsky,[3] and S. Hofferberth[1,†]

[1]5. Phys. Inst. and Center for Integrated Quantum Science and Technology, Universität Stuttgart, Pfaffenwaldring 57, 70569 Stuttgart, Germany
[2]Institute for Theoretical Physics III and Center for Integrated Quantum Science and Technology, Universität Stuttgart, Pfaffenwaldring 57, 70569 Stuttgart, Germany
[3]School of Physics and Astronomy, University of Nottingham, Nottingham, NG7 2RD, United Kingdom

SUPPLEMENTARY NOTE 1: PHOTON PROPAGATION IN THE PRESENCE OF A RYDBERG EXCITATION

For the sake of simplicity we explain our general method explicitly considering the $|50S_{1/2}, 48S_{1/2}\rangle$ pair state and angle $\theta = 0$ between the interatomic axis and the quantization axis. Our model system is a one-dimensional gas of atoms, whose electronic levels are given in Fig. 1(b) in the main text. The photon field $\hat{\mathcal{E}}(z)$ resonantly couples the groundstate $|g\rangle$ with the excited state $|e\rangle$, while 2Ω denotes the Rabi frequency of the control laser field coupling the $|e\rangle$ state with the Rydberg state $|S^{(\mathrm{s})}\rangle$. Following Ref. [1–3], we introduce operators $\hat{P}^\dagger(z)$ and $\hat{S}^\dagger(z)$ which generate the atomic excitations into the $|e\rangle$ and $|S^{(\mathrm{s})}\rangle$ states, respectively, at position z. In addition, comparing to Ref. [1–3] we include a more complex atomic level structure of the source and the gate excitations by defining $\hat{\mathcal{P}}^\dagger(z)$, $\hat{\mathcal{Z}}^\dagger(z)$ and $\hat{\mathcal{B}}^\dagger(z)$ which create excitations into $|P^{(\mathrm{s})}\rangle$, $|S^{(\mathrm{g})}\rangle$ and $|P^{(\mathrm{g})}\rangle$ states, respectively. All the operators $\hat{O}(z) \in \{\hat{\mathcal{E}}(z)\,, \hat{P}(z)\,, \hat{S}(z)\,, \hat{\mathcal{P}}(z)\,, \hat{\mathcal{Z}}(z)\,, \hat{\mathcal{B}}(z)\}$ are bosonic and satisfy the equal time commutation relation, $[\hat{O}(z), \hat{O}^\dagger(z')] = \delta(z - z')$.

The microscopic Hamiltonian describing the propagation consists of three parts: $\hat{H} = \hat{H}_\mathrm{p} + \hat{H}_\mathrm{ap} + \hat{H}_\mathrm{a}$. The first term describes the photon propagation in the medium and is defined as

$$\hat{H}_\mathrm{p} = -ic \int dz \hat{\mathcal{E}}^\dagger(z)\partial_z \hat{\mathcal{E}}(z), \tag{1}$$

with the speed of light in vacuum c, and we set $\hbar = 1$ throughout this work. The atom-photon coupling is described by

$$\hat{H}_\mathrm{ap} = \int dz \;\Big[\; g\hat{\mathcal{E}}(z)\hat{P}^\dagger(z) + \Omega\hat{S}^\dagger(z)\hat{P}(z) + g\hat{P}(z)\hat{\mathcal{E}}^\dagger(z) + \Omega\hat{P}^\dagger(z)\hat{S}(z) - \; i\gamma\hat{P}^\dagger(z)\hat{P}(z) - i\gamma_s\hat{S}^\dagger(z)\hat{S}(z) - i\gamma_p\hat{\mathcal{P}}^\dagger(z)\hat{\mathcal{P}}(z)\Big], \tag{2}$$

where 2γ is the decay rate of the e-level, while g is the collective coupling of the photons to the matter. The interaction between Rydberg levels is described by

$$\hat{H}_\mathrm{a} = \int dz' \int dz \left[\hat{\mathcal{P}}^\dagger(z)\mathcal{B}^\dagger(z')V(z-z')\hat{\mathcal{Z}}(z')\hat{S}(z) + \frac{\Delta_D}{2}\hat{\mathcal{P}}^\dagger(z)\hat{\mathcal{B}}^\dagger(z')\hat{\mathcal{B}}(z')\hat{\mathcal{P}}(z) + \mathrm{H.c.}\right], \tag{3}$$

where $V(z) = C_3/z^3$ is the dipolar interaction potential and Δ_D the Förster defect. Note, that for the experimental parameters $C_3 \gg C_3'$ and therefore it is sufficient to include in the interaction Hamiltonian only the C_3/z^3 coupling term. In addition, it follows that hopping of excitations is quenched, and therefore the $|S^{(\mathrm{g})}\rangle$ excitation is at a fixed position. Then, the description of a single photon propagation requires four components of the wave function: $\mathcal{E}\mathcal{Z}(z,t)$, $P\mathcal{Z}(z,t)$, $S\mathcal{Z}(z,t)$ and $\mathcal{P}\mathcal{B}(z,t)$, which denote the probability of finding the source excitation in $\mathcal{E}, |e\rangle\,, |S^{(\mathrm{s})}\rangle$ or $|P^{(\mathrm{s})}\rangle$ state at position z and the gate excitation in $|S^{(\mathrm{g})}\rangle$ or $|P^{(\mathrm{g})}\rangle$ state at the position z_j. The Schrödinger equation reduces to

$$\partial_t \mathcal{E}\mathcal{Z}(z,t) = -c\partial_z \mathcal{E}\mathcal{Z}(z,t) - igP\mathcal{Z}(z,t), \tag{4}$$

$$\partial_t P\mathcal{Z}(z,t) = -\gamma P\mathcal{Z}(z,t) - ig\mathcal{E}\mathcal{Z}(z,t) - i\Omega S\mathcal{Z}(z,t), \tag{5}$$

$$\partial_t S\mathcal{Z}(z,t) = -\gamma_s S\mathcal{Z}(z,t) - iV_j(z)\mathcal{P}\mathcal{B}(z,t) - i\Omega P\mathcal{Z}(z,t), \tag{6}$$

$$\partial_t \mathcal{P}\mathcal{B}(z,t) = -\gamma_p \mathcal{P}\mathcal{B}(z,t) - iV_j(z)S\mathcal{Z}(z,t) - i\Delta_D\mathcal{P}\mathcal{B}(z,t), \tag{7}$$

where $V_j(z) = V(z - z_j)$. We solve the above set of coupled equations (4-7) via Fourier transform in time, which leads to the equation for the photon field:

$$\left(-ic\partial_r - \frac{g^2 \left(V_{\text{ef}}^j(r) - \omega - i\gamma_s \right)}{-i\gamma\omega + (\gamma - i\omega)\gamma_s - \omega^2 + \Omega^2 - V_{\text{ef}}^j(r)(\omega + i\gamma)} - \omega \right) \mathcal{E}\mathcal{Z}(r,\omega) = 0, \tag{8}$$

with

$$V_{\text{ef}}^j(r) = \frac{C_3^2}{\Delta_D - \omega - i\gamma_p} \frac{1}{(r - r_j)^6}. \tag{9}$$

In the limit of $\gamma_s, \gamma_p \ll \Omega, \gamma$, these expressions simplify to the equations (3) and (4) from the main part of the manuscript.

The equation for the $\mathcal{E}$-field can be generalized to the second pair of states $|66S_{1/2}, 64S_{1/2}\rangle$ by redefining the expression for $V_{\text{ef}}^j(r)$ to

$$V_{\text{ef}}^j(r) = \sum_\alpha \frac{C_{3,\alpha}^2}{\Delta_D^\alpha - \omega - i\gamma_p} \frac{1}{(r - r_j)^6} \tag{10}$$

where we sum over all relevant pairs of states α, which for $\theta = 0$ are

$$\begin{aligned} \alpha \in \quad & \{|65P_{1/2}, m_J = 1/2, 64P_{3/2}, m_J = 1/2\rangle, |65P_{1/2}, m_J = -1/2, 64P_{3/2}, m_J = 3/2\rangle, \\ & |65P_{3/2}, m_J = 1/2, 64P_{1/2}, m_J = 1/2\rangle, |65P_{3/2}, m_J = 3/2, 64P_{1/2}, m_J = -1/2\rangle\}. \end{aligned}$$

* h.gorniaczyk@physik.uni-stuttgart.de
† s.hofferberth@physik.uni-stuttgart.de

SUPPLEMENTARY REFERENCES

[1] Gorshkov, A. V., Otterbach, J., Fleischhauer, M., Pohl, T. & Lukin, M. D. Photon-Photon Interactions via Rydberg Blockade. *Phys. Rev. Lett.* **107**, 133602 (2011).
[2] Peyronel, T. *et al.* Quantum nonlinear optics with single photons enabled by strongly interacting atoms. *Nature* **488**, 57–60 (2012).
[3] Bienias, P. *et al.* Scattering resonances and bound states for strongly interacting Rydberg polaritons. *Phys. Rev. A* **90**, 053804 (2014).
